# *Parachorius semsanganus* sp. n. (Coleoptera, Scarabaeidae, Scarabaeinae) from Laos and its significance in the phylogeny of Oriental Deltochilini

**DOI:** 10.3897/zookeys.111.1221

**Published:** 2011-06-22

**Authors:** S. Tarasov, D. Keith

**Affiliations:** 1 Department of Entomology, Natural History Museum of Denmark/University of Copenhagen, Zoological Museum, Universitetsparken 15, DK-2100 Copenhagen, Denmark; 2 Institute of Natural Science, Kaluga State University, Stepana Razina str. 26, Kaluga 248023, Russia; 3 Muséum des Sciences Naturelles et de Préhistoire, 5 bis, boulevard de la Courtille, 28000 Chartres, France

**Keywords:** Deltochilini, *Parachorius*, *Cassolus*, new species, Laos, “morphological link”

## Abstract

The new species *Parachorius semsanganus* **sp. n.** is described from Laos. This enigmatic Oriental deltochiline represents a “morphological link” between *Parachorius* and *Cassolus* by sharing characters of the two genera. The fact that *Parachorius semsanganus* cannot be unequivocally placed in either of these two genera stresses some more general problems of the current classification of *Parachorius* and *Cassolus*. Such problems can be solved only in the course of phylogenetic analysis, the need of which is briefly outlined.

## Introduction

The dung beetle tribe Deltochilini Lacordaire, 1856 (= Canthonini Lansberge, 1874, synonymy according to Bouchard et al. (2011)), displays a Gondwanian distribution and is the most genus-rich tribe of the Scarabaeinae. It comprises 873 species grouped into 91 genera that constitute 40% of the entire generic diversity of the subfamily Scarabaeinae (Davis et al. 2008). However, the monophyly of the tribe is doubtful according to a revision of African genera (Scholtz and Howden 1987), and it appears polyphyletic in the morphology-based and molecular phylogenies (Philips et al. 2004; Ocampo and Hawks 2006; Monaghan et al. 2007; Sole and Scholtz 2010). Such ambiguity highly complicates the diagnosis of the tribe and thus makes a consensus about its generic composition impossible at the moment.

The Oriental genus *Parachorius* Harold, 1873 comprises six species and, according to the last monographic study (Balthasar 1963), belongs to the tribe Pinotini Kolbe, 1905, which is a synonym of the Ateuchini Laporte, 1840 (Smith 2006; Bouchard et al. 2011). Vaz-de-Mello (2007; 2008) places *Parachorius* within the tribe Coprini Leach, 1815 based on morphological phylogenetic analysis of Ateuchini and related taxa. By contrast, another preliminary morphology-based phylogeny of Oriental Deltochilini using aedeagal and somatic characters (Tarasov, unpublished) strongly suggests that *Canthon* Hoffmannsegg, 1817 (the most speciose genus of the Deltochilini), *Parachorius*, and another Oriental deltochiline genus *Cassolus* Sharp, 1875, form a monophyletic group. Therefore, here, we treat *Parachorius* as a deltochiline. Based on study of external morphological characters within the tribe Deltochilini, *Parachorius* seems to be most closely related to the genus *Cassolus*. The genus *Cassolus* includes nine species that are morphologically very heterogeneous. Such morphological variation, especially when compared with the morphology of the known species of *Parachorius*, may indicate a nested position of the latter genus within *Cassolus* (Tarasov, unpublished).

Whilst surveying recent scarab collections from Laos deposited at the Naturhistorisches Museum in Basel, Switzerland (NHMB), we discovered a very interesting deltochiline species displaying a mixed character set between *Parachorius* and *Cassolus*. This species cannot be unequivocally placed in either genus using current taxonomic concepts of these taxa (Balthasar 1963). However, we tentatively place this new species in the genus *Parachorius*.

Discovery of this species supports the above mentioned evidence for the close relationship between *Cassolus* and *Parachorius* as well as our provisory placement of *Parachorius* within the Deltochilini. Its description, provided here, enables its incorporation in the upcoming phylogenetic analysis of the entire generic complex.

## Material and methods

**Figures 1–5.**
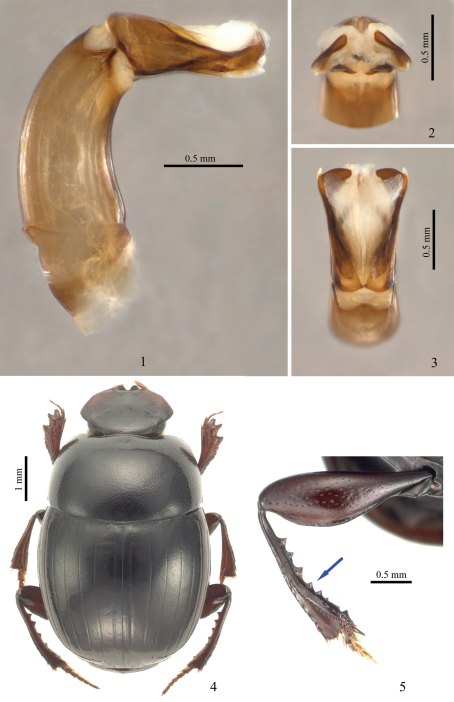
Morphological features of *Parachorius semsanganus* **sp. n.**: **1–3** paratype, aedeagus **1** aedeagus lateral view **2** aedeagus apical view **3** aedeagus dorsal view **4** male holotype, habitus **5** male paratype, hind leg, arrow indicates teeth on inner tibial margin.

All photos were taken with a digital camera attached to a dissecting microscope (Leica MZ16A). Male aedeagi in Figs 1–3 were photographed in glycerin. First, the dissected aedeagus was macerated in 10% solution of KOH for several hours and then rinsed with distilled water. Finally, the aedeagus was placed in glycerin for taking pictures and subsequent storage.

All the material used in this study is housed in the NHMB.

## Species description

### Parachorius semsanganus

Tarasov & Keith sp. n.

urn:lsid:zoobank.org:act:E79FA444-725E-40E6-986A-9FB48B7D232A

http://species-id.net/wiki/Parachorius_semsanganus

Figs 1–5

#### Type locality.

Laos, Xieng Khouang prov., Phou Sane Mt.

#### Material examined.

*Holotype* (NHMB), male bearing the following labels:

LAOS-NE, Xieng Khouang prov., 19°38.20'N, 103°20.20'E, Phonsavan (30 km NE): PHOU SANE Mt., 1420 m, 10.-30.v.2009, D. Hauck leg.

NHMB Basel, NMPC Prague Laos 2009 Expedition: M. Brancucci, M. Geiser, Z. Kraus, D. Hauck, V. Kubáň

HOLOTYPE Parachorius semsanganus S. Tarasov & D. Keith det. 2011

*Paratypes.* 9♀, same data as holotype; 2♂, same data as holotype but Z. Kraus leg.; 4♂, 3♀, LAOS-NE, Xieng Khouang prov., 19°37'N, 103°20'E; 19°38'N, 103°20'E, 30 km NE Phonsavan: Ban Na, Lam Phou Sane Mt., 1300–1500 m, 10.-30.v.2009, M. Brancucci leg.

#### Description.

Oval, convex, black, entirely shiny; mouthparts, antennae and legs red-brown. Dorsal body side covered with two types of punctures: larger (normal) punctures and very tiny punctures (which can be observed only under higher magnification of 40x or more) Dorsal and ventral body sides glabrous. Length 8.2–10.6

##### Male (Fig. 4).

Head flat, punctation fine; anterior margin notched medially; notch delimited by 2 prominent triangular obtuse teeth; clypeus laterad of each tooth very slightly notched; eyes completely divided by canthus into lower and upper lobes; lower lobes significantly larger than upper ones; genae and clypeus not distinctly separated from frons; genae rounded and protruding; antennae with 9 segments, antennal club with 3 segments.

Pronotum broadly trapezoidal, punctation fine, separated by 1–2 puncture diameters on disc, becoming slightly denser laterally. Lateral margins of pronotum flattened, arcuate, widest near base; lateral and anterior side marginate, posterior side not marginate; anterior angles obtuse; posterior angles rounded. Prothoracic fovea excavated, delimited by ridge reaching propleural lateral margin.

Elytra with eight striae, sublateral carina forming pseudepipleuron beyond eighth stria; epipleura narrow; interstriae flat with sparse, fine punctation.

Protibiae with three outer teeth; 1st tooth slightly thicker than two others; inner margin with two vertical teeth underneath, located approximately opposite to 2nd and 3rd outer teeth; protibial apical spur acute, long, reaching middle or apical portion of 3rd tarsal segment; sometimes protibial teeth and apical spur abraded.

Metafemoral posterior margin with keel bearing indistinct and slight serration on top (Fig. 5). Metatibiae slightly curved, conspicuously denticulate on inner margin (Fig. 5, indicated with arrow); teeth are abraded in some specimens.

Pygidium with rather coarse, uniform, dense punctation.

Aedeagus (Figs 1–3) with converging, spatulate apices of parameres.

##### Female

Similar to male but with the 1st protibial outer tooth slightly thinner than in males; metafemoral posterior margin not serrate; metatibial inner margin not denticulate.

##### Variation.

All specimens of the type series look very similar to each other. Some variation may be observed in the shape of teeth on the metatibial inner margin, which are less expressed in some males due to abrasion.

##### Holotype (Fig. 4).

The holotype specimen lacks the tarsus of the right middle leg.

#### Differential diagnosis.

The new species is quite distinct among all other known species of *Parachorius* and *Cassolus*. It can be easily separated from them by the following unique set of character states: clypeus near outer side of each clypeal tooth very slightly notched, metatibial inner margin with large teeth (Fig. 5, arrowed), and aedeagus with spatulate apices which are largely bent inward (Figs 1–3).

#### Distribution and ecology.

The species is known from only 16 specimens of the type series collected across a range of altitudes between 1300–1500 m on Phou Sane Mt. of Xieng Khouang province in Laos.

#### Etymology.

The name of the new species is derived from the Latinized Lao words “syam” – link and “sanga” – spectacular. Its meaning “spectacular link” refers to the fact that this species represents a “morphological link” between the genera *Parachorius* and *Cassolus*.

#### Taxonomic notes.

Based on taxonomic concepts of the most recent monographic study dealing with *Parachorius* and *Cassolus* (Balthasar 1963), the morphological differences between these two taxa can be summarized as follows: *Cassolus* are normally smaller than *Parachorius*; the clypeus near the outer side of each clypeal tooth is usually deeply notched in *Cassolus* and not notched in *Parachorius* (very slightly notched in *Parachorius semsanganus* sp. n.); the metatibiae are curved in *Cassolus* and more or less straight in *Parachorius* (slightly curved in *Parachorius semsanganus* sp. n.); some *Cassolus* species have denticles on the inner metatibial margin, whereas the metatibial margin of *Parachorius* is not at all denticulate (distinctly denticulate in *Parachorius semsanganus* sp.n.). As can be seen from this combination of characters, *Parachorius semsanganus* sp. n.is similar, on the one hand, to *Parachorius* and on the other hand to *Cassolus* (in particular to *Cassolus gotoi* Masumoto, 1986). A robust justification of the taxonomic placement of *Parachorius semsanganus* sp. n. requires an extensive phylogenetic analysis embracing both *Parachorius* and *Cassolus*. Such an analysis is currently in preparation and it may, in particular, result in the synonymy of *Parachorius* and *Cassolus*. Therefore to avoid potential nomenclatural changes in the future, we place the new species in the earlier described genus *Parachorius*.

## Supplementary Material

XML Treatment for Parachorius semsanganus
